# Prevalence of cancer susceptibility variants in patients with multiple Lynch syndrome related cancers

**DOI:** 10.1038/s41598-021-94292-4

**Published:** 2021-07-20

**Authors:** Yoon Young Choi, Su-Jin Shin, Jae Eun Lee, Lisa Madlensky, Seung-Tae Lee, Ji Soo Park, Jeong-Hyeon Jo, Hyunki Kim, Daniela Nachmanson, Xiaojun Xu, Sung Hoon Noh, Jae-Ho Cheong, Olivier Harismendy

**Affiliations:** 1 Department of Surgery, CHA University School of Medicine, Pocheon-si, Korea; 2 Department of Surgery, Yonsei University Health System, Yonsei University College of Medicine, 50 Yonsei-ro, Seodaemun-gu,, Seoul, 120-752 Korea; 3 Yonsei Biomedical Research Institute, Yonsei University Health System, Yonsei University College of Medicine, Seoul, Korea; 4 Department of Pathology, Yonsei University Health System, Yonsei University College of Medicine, Seoul, Korea; 5 Hereditary Cancer Clinic, Yonsei University Health System, Yonsei University College of Medicine, Seoul, Korea; 6 Department of Laboratory Medicine, Yonsei University Health System, Yonsei University College of Medicine, Seoul, Korea; 7 Department of Medicine, Yonsei University Health System, Yonsei University College of Medicine, Seoul, Korea; 8 Moores Cancer Center and Division of Biomedical Informatics Department of Medicine, University of California San Diego School of Medicine, 3855 Health Sciences Dr, La Jolla, CA 92037 USA; 9 Department of Family Medicine and Public Health, University of California San Diego School of Medicine, San Diego, CA USA; 10 Department of Medicine, University of California San Diego School of Medicine, San Diego, CA USA; 11 Bioinformatics and Systems Biology Graduate Program, University of California San Diego School of Medicine, San Diego, USA

**Keywords:** Cancer genetics, Cancer genomics, Clinical genetics

## Abstract

Along with early-onset cancers, multiple primary cancers (MPCs) are likely resulting from increased genetic susceptibility; however, the associated predisposition genes or prevalence of the pathogenic variants genes in MPC patients are often unknown. We screened 71 patients with MPC of the stomach, colorectal, and endometrium, sequencing 65 cancer predisposition genes. A subset of 19 patients with early-onset MPC of stomach and colorectum were further evaluated for variants in cancer related genes using both normal and tumor whole exome sequencing. Among 71 patients with MPCs, variants classified to be pathogenic were observed in 15 (21.1%) patients and affected Lynch Syndrome (LS) genes: *MLH1* (n = 10), *MSH6* (n = 2), *PMS2* (n = 2), and *MSH2* (n = 1). All carriers had tumors with high microsatellite instability and 13 of them (86.7%) were early-onset, consistent with LS. In 19 patients with early-onset MPCs, loss of function (LoF) variants in *RECQL5* were more prevalent in non-LS MPC than in matched sporadic cancer patients (OR = 31.6, 2.73–1700.6, *p* = 0.001). Additionally, there were high-confidence LoF variants at *FANCG* and *CASP8* in two patients accompanied by somatic loss of heterozygosity in tumor, respectively. The results suggest that genetic screening should be considered for synchronous cancers and metachronous MPCs of the LS tumor spectrum, particularly in early-onset. Susceptibility variants in non-LS genes for MPC patients may exist, but evidence for their role is more elusive than for LS patients.

## Introduction

The identification of inherited DNA variants in cancer predisposition genes is clinically important for both cancer prevention and treatment as it can reduce cancer morbidity and mortality in individuals carrying pathogenic variants^1^. The prevalence of pathogenic variants in cancer predisposition genes is lower than 1% of the healthy population and 5–8% in patients diagnosed with cancer^2–4^. Pathogenic variants in cancer predisposition genes are therefore rare. On the other hand, wide-spread surveillance screening may result in false positive findings or findings of uncertain significance, yielding to increased anxiety and unnecessary or ineffective clinical procedures. Thus, continued research supporting more effective and precise genetic screening guidelines is needed^1^.

Multiple primary cancers (MPCs), referring to two or more histologically distinct cancers diagnosed in one individual, is gradually recognized as an important medical problem with a reported frequency of 2–17% in the cancer population^5,6^. Along with early-onset cancer, MPC have been regarded as high-risk of heritability^7^ and those patients therefore represent excellent candidates for genetic screening. However, limited data is available for the prevalence and type of pathogenic variants in the cancer predisposition genes of these patients.

Gastric cancer (GC) and colorectal cancer (CRC) are the two most common cancers in Korea, with 5-year survival up to 70%^8^ and their combination is the most common instance of MPCs, representing ~ 2% of Korean GC or CRC cases^9,10^. About 15% of each of the cancer types can be characterized by elevated microsatellite instability (MSI) caused by mismatch repair (MMR) deficiency^11–15^. Together with Endometrial cancer (EC)—another cancer characterized by MSI^16^—GC and CRC are the main cancer types associated with Lynch Syndrome (LS), caused by pathogenic variants in MMR genes, resulting in up to an 80% lifetime risk of cancers^17,18^. In particular, 2–5% of CRC and EC, as well as ~ 15% of early-onset CRC are thought to be associated with LS^19,20^. MPCs with cancer types in the spectrum of LS therefore represent some of the strongest candidates for genetic screening, as the prevalence of LS among MPC is unknown.

Here we present the results of the genetic screening of 71 patients with MPC of the stomach, colorectal and endometrium. We evaluate the distribution and prevalence of pathogenic variants in 65 cancer predisposition genes, including LS susceptibility genes, as a function of age and clinicopathological features. We further characterize inherited coding variants focusing on DNA repair and cancer related genes in 19 early-onset MPC (eoMPC) patients, proposing candidate MPC susceptibility genes based on the analysis of loss of function (LoF) variants in candidate tumor suppressor genes, their relative prevalence and association with the mutational landscape of the associated tumors.

## Results

### Identification of Lynch Syndrome patients by multigene targeting panel

We investigated the prevalence of LoF variants in cancer predisposition genes using a cohort of 71 patients with MPCs. Fifty-four (76.1%) patients were diagnosed with GC and CRC (23–42.6% synchronous) while 14 diagnosed with EC and CRC (N = 13) or GC (N = 1). Three patients had 3 or more types of cancers. Thirty (42.3%) patients were diagnosed with MPC before age 55 and classified as eoMPCs. A family history of cancer was more prevalent in eoMPCs: of the 56 patients for which family history was available, twenty-four (42.9%) patients had two or more first-degree relatives affected with any type of cancer, and 15 of them were eoMPCs (Odds Ratio [OR] = 8.58, 2.21–39.59, *p* < 0.001). Twenty-six (36.6%) patients had one or more MSI-H tumors, a higher proportion than the one observed in single-cancers of the same type (~ 15%)^11–13,16^ (Supplemental File 1, Table S1). This suggests an important contribution of defects in MMR to MPC development. The germline DNA of the patients was sequenced using a multigene targeting panel of 65 cancer predisposition genes. A total of 15/71 (21.1%) patients were found to carry variants classified to be pathogenic or likely pathogenic (P/LP) and all the variants affected LS related genes (Table 1, Table S2 in Supplemental File 1): *MLH1* (n = 10), *MSH6* (n = 2), *PMS2* (n = 2), and *MSH2* (n = 1), a distribution comparable to the previous reports^17^**.** There was one patient with multi-locus inherited neoplasia alleles syndrome (MINAS) harboring germline pathogenic variants in both *MLH1* and *BRCA1* gene^21^. One of recurrent P/LP variants of *MLH1* was c.1758dupC, a common variant in Korean LS patients^22^. There was a P/LP variant in *MSH2* (c.942 + 3A > T) that was reported as a frequent de novo mutation and founder variant in Newfoundland^23,24^. in a patient diagnosed with four types of cancer (GC, CRC, EC, and lung cancer). Of the remaining 56 of patients, 42 had VUS altering 43 genes, leaving the contribution of these genes to MPC undetermined.

**Table 1:** Summary of clinicopathologic characteristics of patients with multiple Lynch related primary cancers with pathogenic/likely-pathogenic germline variants by targeted panel.

Case ID	Cancer type	Age at second diagnosis‡	Gene	Nucleotide change	Amino acid change	Mutation type	ACMG criteria	Class†	MSI status
dou_002	CRC/GC	< 55	*MLH1*	c.1721 T > C	p.Leu574Pro	Missense	PM2, PP1, PP2, PP3, PP5	LP	MSI-H/MSI-H
dou_003	GC/CRC	< 55	*MLH1*	c.1758dupC	p.Met587HisfsTer6	Frameshift_insertion	PVS1, PM2	LP	MSI-H/MSI-H
dou_005	CRC/GC	< 55	*MLH1*	c.208-1G > A	NA	Splice_acceptor_variant	PVS1, PM2, PP5	P	MSI-H/MSS
dou_006	GC/CRC	< 55	*MLH1*	c.2041G > A	p.Ala681Thr	Missense	PM2, PM5, PP3, PP5	LP	MSI-H/MSI-H
			*BRCA1*	c.213-1G > A	NA	Splice_acceptor_variant	PVS1, PM2, PP5	P	
dou_011	GC/CRC	< 55	*MLH1*	c.790 + 2 T > A	NA	Splice_donor_variant	PVS1, PM2, PP5	P	MSI-H/MSI-H
dou_016	GC/CRC	< 55	*MLH1*	c.1758dupC	p.Met587HisfsTer6	Frameshift_insertion	PVS1, PM2	LP	MSI-H/MSI-H
dou_017	GC/CRC	< 55	*MLH1*	c.1559-2A > C	NA	Splice	PVS1, PM2, PP5	P	MSI-H/MSI-H
dou_047	CRC/GC	> 55	*MSH6*	c.829G > T	p.Glu277Ter	Splice_acceptor_variant	PVS1, PM2	LP	MSI-H/NA
dou_056	CRC/EC	> 55	*PMS2*	c.1738A > T	p.Lys580Ter	Nonsense	PVS1, PM2, PP5	P	MSI-H/MSS
dou_061	CRC/EC	< 55	*MSH6*	c.3477C > G	p.Tyr1159Ter	Nonsense	PVS1, PM2, PP5	P	NA/MSI-H
dou_062	CRC/EC	< 55	*PMS2*	c.943C > T	p.Arg315Ter	Nonsense	PVS1, PM2, PP5	P	MSI-H/MSI-H
dou_065	CRC/EC	< 55	*MLH1*	c.67G > T	p.Glu23Ter	Nonsense	PVS1, PM2, PP5	P	MSI-H/MSI-H
dou_069	CRC/EC/Klaskin	< 55	*MLH1*	c.67G > T	p.Glu23Ter	Nonsense	PVS1, PM2, PP5	P	MSI-H/MSI-H/MSI-H
dou_070	EC/CRC/GC	< 55	*MLH1*	exon 7–9 deletion	NA	NA	PVS1, PM2	LP	MSI-H/NA/MSI-H
dou_071	CRC/GC/EC/Lung	< 55	*MSH2*	c.942 + 3A > T	NA	Exon loss	PVS1, PM2	LP	MSI-H/MSI-H/NA/MSI-H

*The order of MSI/MMR status is matched to cancer type.

^†^LP: likely pathogenic, P: Pathogenic.

^‡^The age of the patients is described as a range to preserve the anonymity of the patients as recommended by institutional review board.

ACMG, the American College of Medical Genetics and Genomics.

### Characteristics of LS patients

**Figure 1:**
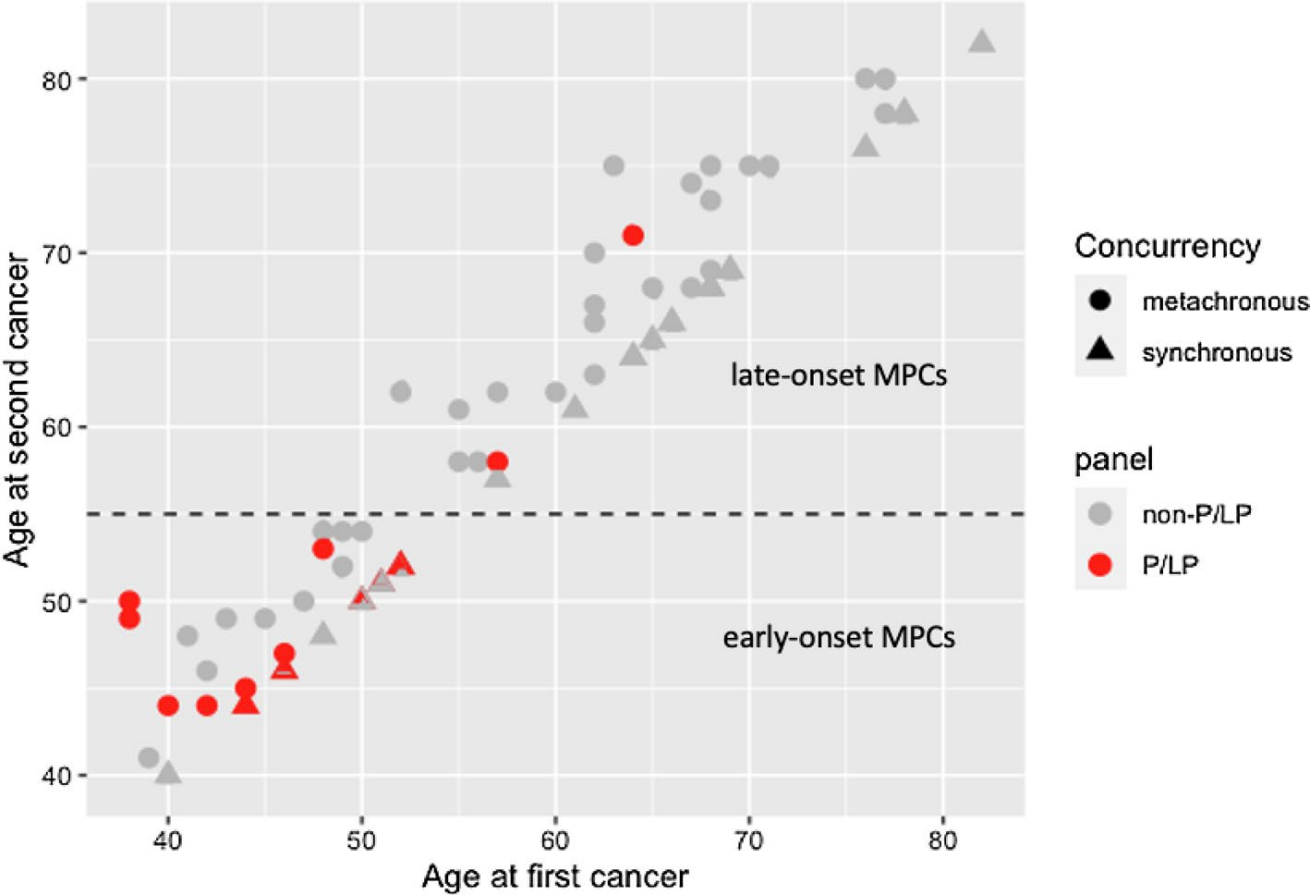
A scatter plot between age of first and second cancer by with/without pathogenic or likely pathogenic (P/LP) germline variants and concurrency of the tumors in each patient. There were 15 (21.1%) of patients with germline P/LP variants (red colors). Among early-onset multiple primary cancers (MPCs), 43.3% (13/30) patients had P/LP variants while only 4.9% (2/41) patients were related to P/LP variants in late-onset MPCs.

All 15 LS patients had at least one MSI-H tumor in contrast to 11/56 non-LS patients. LS patients were also more likely to have two or more first-degree relatives (9/10, OR = 17.67, 2.12–836.73, *p* = 0.001, Supplemental file 1, Tables S1 and S2). The association of the variants with tumor characteristics were further investigated. The loss of expression of the protein corresponding to the gene with P/LP variants was confirmed in all 19 MSI-H tumors investigated from all LS patients (Supplemental file 1, Table S3)^25^. The LS patients were more likely to be diagnosed with eoMPC (13/15, OR = 14.31, 2.79–144.22, *p* < 0.001, Fig. 1) but equally likely to present with synchronous tumors (6/15, OR = 0.89, 0.23–3.26, *p* > 0.999). This suggests that consistent with Lynch syndrome, LS MPC patients are diagnosed earlier. However, the variants are unlikely to influence the relative timing of the two cancer diagnosis. More importantly, these results showed that the majority of MPC patients do not have pathogenic variants known to cause LS. Even restricting to 13 patients with eoMPCs and strong family history of cancer (two or more first-degree relatives over two successive generations), cancer predisposition genes were not altered in nearly half of the patients (6/13, Table S2 in the Supplemental file 1). The likely genetic cause of MPC in these patients remains undetermined.

### Identification of candidate cancer predisposition genes in early-onset MPC

To identify additional variants and genes underlying a possible cancer susceptibility in non-LS patients, we sequenced the whole exome of 19 eoMPC patients with gastric and colorectal cancer which is the most common combination of MPCs in Korea^9,10^. Seven of the patients were LS patients, as determined above, and used as controls for the cancer predisposition genes discovery process. We identified 111,842 high-confidence variants across all patients, of which 3,211 were rare (MAF < 0.01) in East-Asian population and predicted to be damaging, affecting 2,675 genes. We investigated a set of 382 genes (referred to as cancer genes) involved in DNA repair, cancer susceptibility and progression (Supplemental file 1, Table S4)^26^. Of those, 66 were altered by 82 variants, including 7 in the *MLH1* gene of the LS patients, therefore confirming the panel sequencing results and validating the initial WES variant calling and filtering approach.

For a given candidate cancer predisposition genes, we expect that the prevalence of LoF alterations observed in eoMPC cohort would be higher than in a matching sporadic cancer cohort. Despite some LS phenotypes being reported^26^, TCGA gastric and colorectal cancer patients are mainly from sporadic cancers. We selected 70 of these patients with East-Asian ancestry to closely match the Korean ancestry of eoMPC cohort and reduce spurious findings resulting from population differences. In these patients, we identified a total of 87 LoF variants affecting 47/382 cancer genes. We confirmed that LoF variants in *MLH1* were more prevalent in LS patients than in TCGA patients (7/7 vs 2/70 *p* < 0.001 Table 2, Table S5 in the Supplemental file 1). Importantly, 5/7 of the *MLH1* LoF variants in LS patients were predicted to be high-confidence LoF variants, indicating that most, but not all, known pathogenic variants can be identified through this approach. *RECQL5* was the only gene significantly more altered in non-LS patients than in TCGA patients (4/12 vs 1/70, OR:31.66, 2.73–1700.6, *p* = 0.0013, Table 2). In particular two of these patients had the same *RECQL5* variant (p.R441Q which is not seen in the TCGA patients (*p* = 0.02, Table S5in the Supplemental file 1) The variant is rare, with a global minor allelic fraction of 3.6 × 10^–4^. Interestingly 89 of the 101 observed minor alleles (out of 278,974 total alleles observed) in the gnomAD dataset^27^ belong to the East-Asian population, suggesting a possible Korean-specific effect.

**Table 2:** Genes affected by Loss of function variants in two or more patients with eoMPC at stomach and colon.

Gene	N mutated cases				*p*-value* (TCGA)	*p*-value + (TCGA)
	Non-Lynch (n = 12)	Lynch (n = 7)	Total (n = 19)	TCGA_EAS (n = 70)		
*MLH1*	0	7	7	2	> 0.999	** < 0.0001**
*RECQL5*	4	0	4	1	**0.0013**	> 0.999
*EME2*	2	0	2	1	0.0547	> 0.999
*EP300*	2	0	2	1	0.0547	> 0.999
*MUTYH*	2	0	2	1	0.0547	> 0.999
*MSH3*	2	0	2	2	0.1002	> 0.999
*COL7A1*	2	0	2	4	0.2108	> 0.999
*PTCH1*	2	0	2	7	0.6134	> 0.999

*p* < 0.01 values are in bold.

*Fisher's exact *p*-value for the frequency of each gene between non-lynch and TCGA.

" + "Fisher's exact *p*-value for the frequency of each gene between lynch and TCGA.

### Bi-allelic alterations in corresponding tumors as a guide for cancer predisposition genes identification

According to Knudson’s two-hit model^28^, the wild-type allele of tumor suppressor genes is frequently lost or mutated in the tumor. Such combination of inherited deleterious variants with somatic loss—referred to as bi-allelic alteration—can help prioritize candidate cancer predisposition genes^26,29^. We sequenced the exome of 37 tumors from all 19 patients with eoMPC (Supplemental file 1, Table S7) and identified 35 somatic bi-allelic alterations by LoF mutations (N = 5), loss of heterozygosity (N = 27) or both (N = 3) focusing on candidate germline LoF genes.

The *MLH1* gene was *somatically* altered in 13/15 tumors from LS patients and at least one tumor from each of the 7 LS patient. (Table 3). Importantly, 5/7 *MLH1* variants in LS patients were high-confidence LoF variants. This observation confirms the validity of the investigation of bi-allelic alteration to identify cancer predisposition genes, at least for high-confidence LoF variants in known predisposition genes. In contrast, the variants in the main candidate susceptibility gene in non-LS patients, *RECQL5*, were not high-confidence LoF variant and the genes was not affected in any of the 6 tumors from 4 variant carriers or any of the 22 tumors from all non-LS patients. Expanding the analysis to other candidate genes affected by LoF germline variants in non-LS patients, we identified 17 genes in 10 patients also affected by bi-allelic alterations in one or more tumors. Two were altered via mutations and 15 through LOH. Among these, 2 genes were affected by high-confidence LoF germline variants: *FANCG* (germline splice site variant, c.307 + 1G > C) in gastric tumor of dou_004 and *CASP8* (germline deletion variant, c.658_659del) in colorectal tumor of dou_010 (Table 3). Interestingly, this analysis also revealed somatic alterations of LS genes in absence of inherited LS mutation in a non-LS patient (dou_009) with both of MSI-H tumors in the stomach and colon. Indeed, somatic allelic imbalance was observed in *MLH1* (stomach and colon) and *PMS2* (stomach only) and the expression of both encoded proteins was lost from both tumors. Loss of one allele in the tumor would likely not be sufficient to impair MMR, raising the possibility of undetected germline LoF variants in these LS genes in this patient, perhaps through alterations not easily detected by WES^30^. There was one tumor (colon cancer of dou_005) with double somatic mutations^31^ in MMR genes (c.582-591del at *MLH1* and c.493delT at *PMS2*, variant allele fraction was 0.186 and 0.188, respectively) and the patient harbors germline pathogenic variant at *MLH1*. The details of LoF germline variants and somatic alteration of the corresponding tumors were described in Table S6 (Supplementary file 1).

**Table 3:** Summary of candidate loss-of-function germline candidate genes and the status of loss of heterozygocity of corresponding tumors.

Patient group	Case ID	Genes with germline LoF variants (1)	N tumors (N sequenced)	Tumor MSI status (GC/CRC)	Bi-allelic alterations in at least one tumor (1)
Lynch group	dou_002	MLH1*, FANCL, RAD54L, RFC3, TSC2, FANCA, ARID1B, POLN	2 (1)	MSI-H/MSI-H	*MLH1*
	dou_003	MLH1*, POLL*, SMO*	3 (3)	MSI-H/MSI-H/MSI-H	MLH1**, SMO**
	dou_005	MLH1*, FANCI, UNG, POLN, RFC3, ARID1A, FANCI	2 (2)	MSS/MSI-H	*UNG, POLN, *MLH1**, ARID1A**
	dou_006	*MLH1, ****BRCA1****, CBL, RAD50, UVSSA*	2 (2)	MSI-H/MSI-H	*MLH1, RAD50*
	dou_011	MLH1*, ARID2, ERCC2, EGFR, TMEM127, RHBDF2	2 (2)	MSI-H/MSI-H	*ARID2, RHBDF2, *MLH1**
	dou_016	MLH1*, DCLRE1C, ERCC6, JAK3	3 (3)	MSI-H/MSI-H/MSI-H	MLH1***
	dou_017	MLH1*, GNAS	2 (2)	MSI-H/MSI-H	MLH1**
Non-Lynch group	dou_001	*MUTYH, PBRM1, PTCH1, ABL1*	2 (2)	MSS/MSS	*MUTYH, PBRM1*
	dou_004	*CDH1, FANCI, ****FANCG****, FANCM, RAD50, ****MSH3***	2 (2)	MSS/MSS	*CDH1, FANCI, ****FANCG****, FANCM*
	dou_007	BRCA2, MUTYH, EME2, MBD4, RECQL5*, WRN, COL7A1	2 (1)	MSS/NA	
	dou_008	AXIN1, COL7A1, RECQL5*, MSH4, PTCH1, NEIL3, KLF4	2 (2)	MSS/MSS	*COL7A1*
	dou_009	NOTCH2, REV3L, RECQL5*, SMARCA4, EP300, DOCK8	2 (2)	MSI-H/MSI-H	*SMARCA4*
	dou_010	***CASP8****, EME2, NOTCH1*	2 (2)	MSS/MSS	***CASP8***
	dou_012	*POLM, EME1*	2 (2)	MSS/MSS	*EME1*
	dou_013	*FLCN, CIC, NFATC2IP, XAB2*	2 (2)	MSS/MSS	*FLCN*
	dou_014	EP300, XPC, RECQL5*	2 (1)	MSS/NA	
	dou_015	*RAD52, CHEK2*	2 (2)	MSS/MSS	*RAD52*
	dou_018	*PARG, TET2, NF2, UVSSA, BCOR*	2 (2)	MSS/MSS	*PARG, TET2, UVSSA, NF2*
	dou_019	*MSH2, ERCC6, ALKBH2*	2 (2)	MSS/MSS	*ERCC6*

(1) High confidence LoF prediction in bold.

*Significantly recurrent in eoMPC.

**Bi-allelic alteration was observed in 2 tumors.

***Bi-allelic alteration was observed in 3 tumors.

## Discussion

As expected, the present result confirmed that individuals with MPC, particularly early onset, have a higher likelihood of LS: MMR related P/LP germline variants were observed in 21% of MPC (15/71) and in 43% of eoMPC (13/30) patients, a proportion comparable to ~ 15% of early-onset CRC^20^. The elevated extracolonic cancer risk following colorectal cancer in Lynch syndrome was reported (~ 5 and 40 times of gastric and endometrial cancer risk compared to general population, respectively)^32^, and this study supports those results. Therefore, due to the high prevalence of germline mutations individuals with MPC in the LS tumor spectrum should undergo germline testing, including when MSI status is unknown.

Because MSI-H was considered as a hallmarks of LS, universal screening of all CRC and EC has been recommended^33,34^. In addition, MSI screening for gastric cancers in regions where gastric cancer is highly prevalent like Korea is a way to identify individuals and families who can benefit from germline testing of LS genes, so that surveillance and risk reducing interventions can be undertaken along with cascade testing of family members. At the same time, MSI testing is valuable for other types of solid cancers for other purpose: MSI-H is a biomarker for response to immune checkpoint inhibitors that is a breakthrough for treating advanced solid cancers regardless of its origin^35^. As MSI-H is predictive of LS across a broad tumor spectrum^36^, screening of tumor MSI status in all patients with an initial cancer, especially for LS spectrum tumors and early-onset cancer, and consecutive germline testing for patients with MSI-H tumor will help refine the diagnosis, giving an opportunity to diagnose LS and avoid or detect the second cancer as early as possible.

Genetic analysis of families with high occurrence of cancer using cancer predisposition genes panels or exome sequencing has become standard to identify the underlying cancer susceptibility variants. But penetrance, interaction with environmental factors, and size of the pedigree may affect power of such linkage studies^1,37–39^. Evaluating both germline and somatic alterations is another reasonable approach to find cancer predisposition genes^26,29^. The present results showed that characterizing recurrent LoF genes, truncating variants and loss of heterozygosity are useful to prioritize candidate cancer predisposition genes; any combination of them unambiguously identified germline *MLH1*, a known cancer predisposition genes in LS patients, in multiple patients clinically diagnosed with LS. It suggests that MPC is a strong phenotype to find cancer predisposition genes, therefore this approach would be worthy to be expanded to other combinations of MPCs. However, the types of MPCs would be different by geographical regions or ancestry: region-specific environmental factors can contribute to the oncogenesis and lead to different cancer types within the LS spectrum. For instance, MPCs including GC could be a hallmark of LS in Korea but not in the United States where GC incidence is much lower. This could explain why GC risk in LS may have been underestimated^17,40^. Therefore, geographical and/or ancestry specific cancer epidemiology needs to be accounted for in the genetic screening guidelines.

In non-LS patients with eoMPC, however, evidence of germline susceptibility was more elusive. *RECQL5* was a frequently recurrent gene and variant (p.R441Q) compared to East Asian sporadic cancer population (TCGA). Despite careful matching, some differences between the eoMPC and matched TCGA cohorts remained and could have confounded this observation. Notably, eoMPC patients were all younger than 55 and exclusively of Korean descent, while the TCGA patients included in the analysis were not selected for age and included non-Korean Asians. Importantly, the allele frequency of the *RECQL5* variant was 0.4% of EAS population in gnomAD, and there was no evidence of somatic LOH in the corresponding tumors, suggesting that additional analysis and experiment are required to establish its pathogenicity. *CASP8* and *FANCG* were high-confidence LoF germline gene with bi-allelic alteration in CRC and GC, respectively. *CASP8* encodes a member of caspase family and play a role in apoptosis, and some of its polymorphism have been reported as susceptibility to various cancers including CRC^41–43^. *FANCG* is a gene encoding Fanconi anemia (FA) group G protein, and part of the FA DNA damage repair pathway and in which germline pathogenic variants often predispose to cancers^44–46^. Thus, the *CASP8* and *FANCG* variants identified in these two MPC cases could underlie their disease susceptibility though additional functional studies would be necessary to demonstrate their pathogenicity^47–50^.

Despite evaluating all inherited coding variants in cancer genes and somatic changes in the corresponding tumors, we did not find a clear inherited predisposition that caused multiple cancers in over half of patients with eoMPC, even with strong family cancer history. Not only cancer predisposition genes, but also environmental factors such as smoking and alcohol also increase the risk of cancers^51–53^.

Our study has inherent limitations. The present results did not cover epigenetic changes affecting candidate cancer predisposition genes in tumors, and methylation of the *MLH1* promoter in particular is known to be a mechanism for somatic loss of function in ~ 20% of CRC^54^. Furthermore, we restricted the analysis to 382 well studied cancer genes, more likely to impact cancer susceptibility, therefore leaving the possibility to miss un-expected cancer predisposition genes. The complete analysis of whole exomes to identify rare disease susceptibility variants would indeed be intractable for the cohort under study. Careful analysis of the pedigree and genetic comparison of family members, a typical standard in such genetic susceptibility studies, was not possible due to the absence of family history information in many cases and to the retrospective nature of the study. However, this study is to our knowledge the largest study of patients with MPCs, evaluated using the same multigene targeting panel, and where both germline and multiple tumors DNA were investigated in the highest risk patients.

eoMPC of colorectal, endometrial, and gastric cancer are considerably enriched for LS patients, supporting the genetic screens of related family members as well as enhanced monitoring for younger patients after their first diagnosis. Routine tumor screening of MSI for patients with initial LS related cancers and consecutive germline testing for patients with MSI-H is worthy to diagnose LS and early detection or avoid second cancer. While susceptibility variants in non-LS genes for MPC patients may exist, evidence for their role is more elusive than for LS patients and would require deeper genetic investigation and complementary functional studies.

## Methods

### Population

We performed a retrospective population-based study targeting patients who were treated for two or more cancers in the stomach, colorectum, or endometrium at Severance Hospital, Yonsei University College of Medicine between January 2001 and December 2016. All methods were carried out in accordance with relevant guidelines and regulations. This study was approved by Institutional Review Board (IRB) of Severance hospital of the Yonsei University Health System and by the IRB of the University of California, San Diego (4-2017-0434, 191,543). The IRB of Severance hospital of the Yonsei University Health System waived the requirement for patient informed consent as the study is retrospective by design. The patients were selected using following criteria: (1) two or more cancers were pathologically confirmed, (2) multiple tumors were histologically different, not a metastatic or recurrent tumor from one cancer, (3) normal tissues were available and histologically confirmed for sequencing. DNA was obtained from formalin fixed paraffin embedded (FFPE) normal and tumor tissues. Early-onset MPC (eoMPC) was defined by the diagnosis of a second cancer at age 55 or younger. The clinico-pathologic characteristics of the patients and tumors including age, sex, family history of cancer, MSI or MMR status of tumors were evaluated. For evaluating MSI status, two mononucleotide repeat markers (BAT25 and BAT26) and three dinucleotide repeat markers (D5S346, D2S123, and D17S250) were used by polymerase chain reaction^55^, and MSIsensor was used with cut-off of MSI score > 3.5 in Whole Exome Sequencing (WES) data^56^. To evaluate MMR protein expression, immunohistochemistry (IHC) was conducted in four MMR genes; *MLH1* (ready-to-use, clone M1, Roche, Indianapolis, IN, USA), *MSH2* (ready-to-use, clone G219-1129, Roche), *MSH6* (1:100, clone 44, Cell Marque, Rocklin, CA, USA), and *PMS2* (1:40, clone MRQ28, Cell Marque). An MMR-deficient (dMMR) tumor was defined as a tumor showing loss of expression of any of the four MMR proteins. If a tumor was classified as any one of MSI-high (MSI-H) or deficiency MMR (dMMR) it was considered as MSI-H. The concurrency of tumors was classified as synchronous tumors when the interval between tumors was less than 1 year, otherwise considered as metachronous tumors.

### Germline multigene targeting panel

Germline DNA of patients with MPCs was evaluated using a customized targeted capture sequencing panel (OncoRisk, Celemics, Seoul, Korea) covering all coding sequences and intron–exon boundaries of the coding exons of known 65 cancer predisposition genes (Supplemental file 1, Table S8)^57^. Both structural variants and nucleotide variants were evaluated. The germline variants were classified into pathogenic, likely pathogenic, variant of uncertain significance (VUS) and reported following the guidelines of the American College of Medical Genetics and Genomics 2017^58^. Only pathogenic or likely pathogenic (P/LP) germline variants in cancer predisposition genes were considered in the analysis. Read-depth based detection of structural variants was conducted using the ExomeDepth software. Chromosomal copy number variations (CNVs) detected by ExomeDepth were further crosschecked using our custom pipeline; this retrieved base-level depth-of-coverage for each bam file using SAMtools software and normalized the depths against those of other samples in the same batch. There were no significant pathogenic CNV detected in the studied samples.

### Whole exome sequencing

#### Data generation

WES of normal and tumors was conducted for patients with eoMPCs in the stomach and colon. Genomic DNA was extracted from the confirmed normal and tumor tissues of FFPE. SureSelect sequencing libraries were prepared according to the manufacturer’s instructions (Agilent SureSelect All Exon V6 kit, Santa Clara, CA, USA), and HISEQ2500 sequencing system (Illumina™, San Diego, CA, USA) performed sequencing with read lengths of 2 × 100 bp. The statistics and quality metrics of normal and tumors were described in Supplemental file 1, Tables S7 and S9.

#### Data analysis

The reads were aligned to hg19 reference genome by Burrows-Wheeler Aligner software (BWA 0.7.17) and duplicated reads were removed by Picard (MarkDuplicates) through bcbio-nextgen (v.1.2.3)^59^ For germline and somatic analysis, variants were called by Genome Analysis Toolkit Haplotype joint caller (GATK v3.9) and mutect2^60,61^, respectively. Variants were annotated by refGene using ANNOVAR^62^, CADD scores (CADD13 and CADDindel)^63^, population allelic frequency in ExAC v3.0^64^, and membership to ClinVar^65^.

To discover novel cancer predisposition genes, we focused on high-confidence (TLOD > 12, FS < 10, SEQQ > 60, MQ ≥ 60, STRANDQ > 40, DP > 10), rare (minor allele fraction was < 0.01 in ExAC of Eastern Asian population), and damaging (exonic/splicing, non-synonymous variants with > 20 of CADD score) variants in 382 genes of 11 cancer-relevant pathways^26^, and they were defined as LoF germline variants. High-confidence of LoF variants were predicted by Loss-Of-Function Transcript Effect Estimator (LOFTEE) plugin for Ensembl VEP (v.99) that targeting stop-gained, splice site disrupting, frameshift variants^66,67^. Copy number alterations of tumors compared to normal was estimated using CNVkit (V0.8)^68^ The gene level copy number ratio was calculated as the weighted mean of all bins covered by the whole segment overlapping the gene. Genes had ≥ 3 segments of copy number changes were included, and log2 copy number < − 0.3 was defined as deletion. Somatic allelic imbalance (AI) in tumors of a given heterozygous germline variants were estimated using hapLOHseq^69^. Germline variants of tumors were obtained by GATK haplotype caller and filtering out variants were not observed in germline variants in normal sample. Phase of genotypes were estimated with a companion phasing utility with hapLOHseq. Event prevalence was set 0.1, and AI was defined when posterior probability of being in AI was over 90%. To assess loss of heterozygosity (LOH) of a given heterozygous germline variants in tumors as a secondary hit mechanism of inactivation of cancer predisposition genes^26,28^, we evaluated germline and somatic bi-allelic alteration of corresponding tumors. When there was any one of somatic damaging mutation or AI event in candidate germline cancer predisposition genes, it was considered as bi-allelic alteration.

To compare the frequency of recurrent cancer predisposition genes in patients of eoMPCs to that of the cancer genome atlas (TCGA) cohort, germline data of East-Asian patients (≥ 80% of admixture)^70^ with stomach cancer or colorectal cancer in TCGA cohort was used^26,71^. Variants were filtered for rare and damaged variants similar to LoF germline variants in MPCs cohort. For recurrent variants and genes in eoMPCs cohort, the frequency was compared between eoMPCs cohort and selected TCGA cohort.

### Statistical analysis

Continuous variables were compared by two-sided Mann–Whitney test and categorical variables were compared by two-sided Fisher’s exact test. *P* < 0.05 was considered as statistically significant and R version 3.6.1(R Foundation for Statistical Computing, Vienna, Austria) was used for generating figures and statistical analysis.

## Supplementary Information

Supplementary Information.
